# Case Report: Diabetes in Chinese Bloom Syndrome

**DOI:** 10.3389/fendo.2021.524242

**Published:** 2021-06-09

**Authors:** Mingqun Deng, Miao Yu, Ruizhi Jiajue, Kai Feng, Xinhua Xiao

**Affiliations:** Department of Endocrinology, Key Laboratory of Endocrinology, Ministry of Health, Peking Union Medical College Hospital, Beijing, China

**Keywords:** Bloom syndrome, diabetes, short stature, azoospermia, leptin

## Abstract

Bloom syndrome (BS) is a rare autosomal recessive disorder that causes several endocrine abnormalities. So far, only one BS pedigree, without diabetes, has been reported in the Chinese population. We presented the first case of BS with diabetes in the Chinese population and explored the clinical spectrum associated with endocrine. Possible molecular mechanisms were also investigated. Our study indicated that BS may be one rare cause of diabetes in the Chinese population. We also found a new pathogenic sequence variant in *BLM* (BLM RecQ like helicase gene)(NM_000057.4) c.692T>G, which may expand the spectrum of *BLM* variants.

## Introduction

Bloom syndrome (BS) was first reported by Bloom, a dermatologist, in 1954, and there are currently no more than 300 cases of BS reported worldwide. BS is a rare cause of diabetes, and no cases of diabetes in the Chinese population have been reported. BS can lead to clinical phenotypes including many other endocrine abnormalities, which required attention from endocrinologists. This study aimed to improve the understanding of BS from the perspective of endocrinologists.

## Case Report

The patient was a 19-year-old male. He was born in full-term, with a weight of 1.8 kilograms (kg) and a height of 40 centimeters (cm). Apart from growth failure, no developmental delay was presented. He reached his final height of 143 cm at his 14s and his maximum weight was 42 kg at his 15s. The patient repeatedly experienced infection of the upper respiratory tract during his childhood, and he was diagnosed with “acute type A hepatitis” at his 1.5 years. He was also diagnosed with “cytomegalovirus pneumonia” at 8 years old. The patient showed facial erythema since he was 2 years old. One year ago, the patient developed well-demarcated patchy areas of hypopigmentation and hyperpigmentation. In 2013, when he was 13 years old, the patient started to experience polydipsia, polyphagia, and polyuria. His fasting blood glucose (FBG) was revealed to be 19 mmol/L, and the urinary ketone body was positive. C-peptide level and type 1 diabetes-related auto-antibodies were unknown. Though with 14 units insulin aspart for each meal and 21 units glargine insulin a day, his blood glucose was not controlled well (FBG 8-14 mmol/L, and 2 hours postprandial glucose 10-18 mmol/L). Four years after diabetes onset, his fasting C-peptide (FCP) was 1.70 ng/mL and 2 hours postprandial C peptide (2hCP) was 3.19 ng/mL, with simultaneous FBG 13 mmol/L and 2 hours postprandial glucose (2hPG) 27 mmol/L. Glycated hemoglobin (HbA1c) was 10%. In January 2019, a total of 36 U insulin aspart and insulin glargine 38 U per day still failed to control his glucose. In June 2019, the patient went to our department.

**Figure 1:**
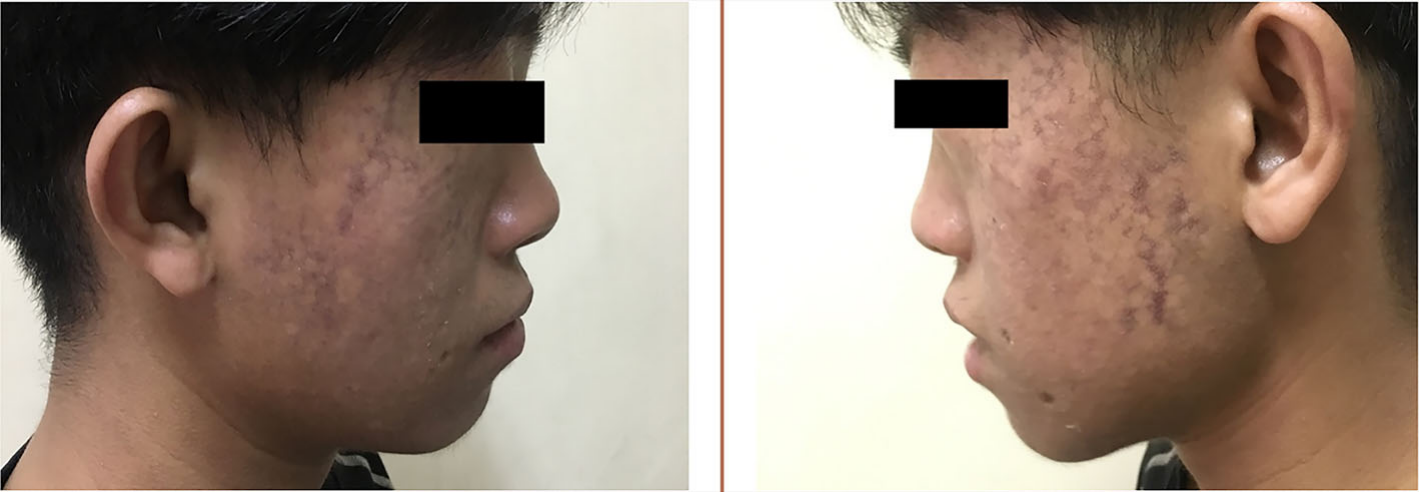
Symmetrical erythema on the patient’s face.

The proband’s parents denied consanguinity. His grandmother was diagnosed with type 2 diabetes (T2D) in her 60s. His father was also diagnosed with T2D when he was 43 years old, with a body mass index (BMI) of 33.1 kg/m^2^. Physical examination revealed a height of 143 cm and a weight of 37 kg (BMI 18.09 kg/m^2^), his waist circumference was only 60 cm. Symmetrical erythema can be seen on his face (**Figure 1**), areas of hypopigmentation and hyperpigmentation scattered. Tanner stage was V, with a length of penis 6 cm and the volume of bilateral testes was 8 ml, respectively. No other abnormalities discovered. BS was suspected and further clinical investigation was implemented.

**Figure 2:**
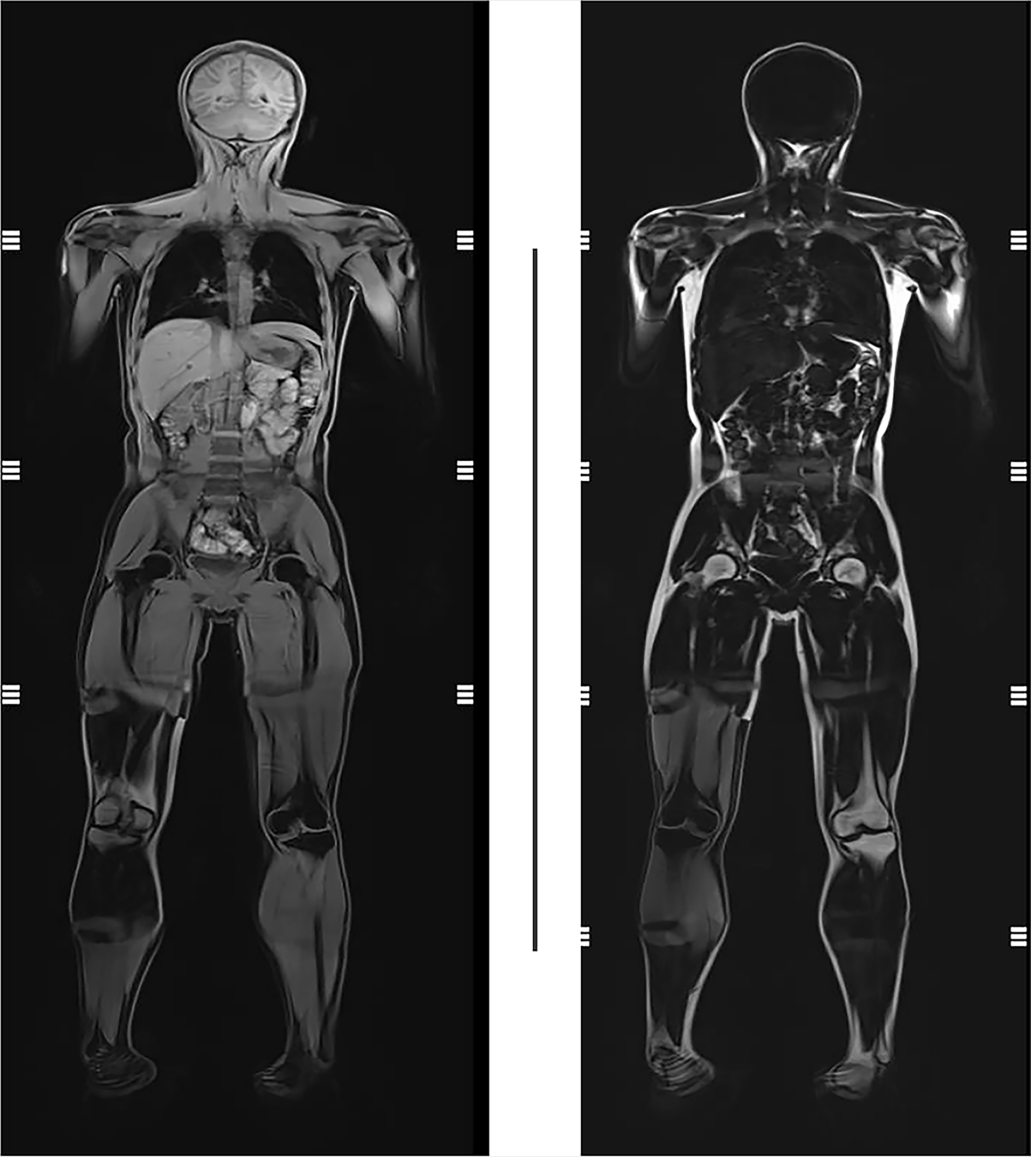
Quantitative MRI of fat of the proband. No reduction or abnormal distribution of fat was presented.

His glycated albumin (GA%) was 22.4% and HbA1c was 10.2%, which suggested poor glycemic control with a high dose of insulin (2 U/kg·d, 74 units per day). FCP was 0.43 ng/mL, and 2hCP was 1.91 ng/mL, while synchronous FBG was 9.3 mmol/L and 2hPG was 15.7 mmol/L, indicating islet dysfunction. Hyperlipidemia did not exist: total cholesterol (TC) 2.85 mmol/L, triglyceride (TG) 1.06 mmol/L, high density lipoprotein cholesterol (HDL-C) 1.06 mmol/L, low density lipoprotein cholesterol (LDL-C) 1.41 mmol/L. However, an abdominal ultrasound indicated fatty liver. The level of leptin was only 0.5 ng/mL. Quantitative MRI of fat did not suggest a reduction or abnormal distribution of fat (**Figure 2**). Metformin 0.5g tid and pioglitazone 30mg qd were added, while the total amount of insulin reduced from 74 units to 64 units per day. His FBG was 5-8 mmol/L and 2hPG was 8-12 mmol/L. Though the patient seemed normal in puberty development, his level of follicle-stimulating hormone (FSH) was as high as 19.36 IU/L, with normal luteinizing hormone (LH) of 7.17 IU/L and testosterone (T) of 4.64 ng/ml. No sperms were detected in his semen. No abnormality of hypothalamic-pituitary-adrenal axis and the hypothalamic-pituitary-thyroid were revealed. Growth hormone (GH) 0.3 ng/ml and insulin-like growth factor 1 (IGF1) 36 ng/ml were normal. Blood parathyroid hormone, calcium and phosphorus were all within the normal range.

This study was approved by the Ethics Committee of PUMCH (Approval number is JS-1233) and informed consent was obtained from the proband and his parents. The genomic DNA of the proband and his parents were extracted from peripheral blood using the DNA extraction kit of Beijing Tiangen Biochemical Technology Co., Ltd. The PCR product was identified by 1.5% agarose gel electrophoresis, purified and sequenced using ABI 3730 automatic sequencer (Applied Biosystems, USA). The results were analyzed by DNAStar6.0 software (Lasergene, USA) subroutine SeqMan. The standard sequence was based on the NCBI database candidate gene reference sequence.

**Figure 3:**
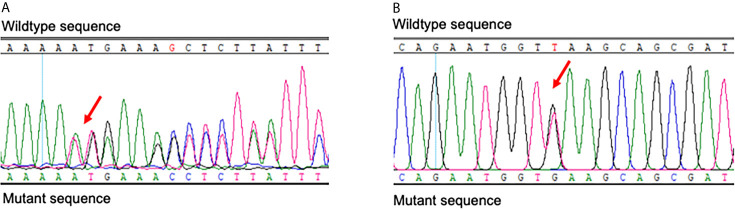
Results of Sanger sequencing of *BLM* of the proband. The red arrows indicate the c.1544delA (p.Asn515Metfs*16) **(A)** and c.692T>G (p.Leu231*) **(B)** heterozygous variants.

**Figure 4:**
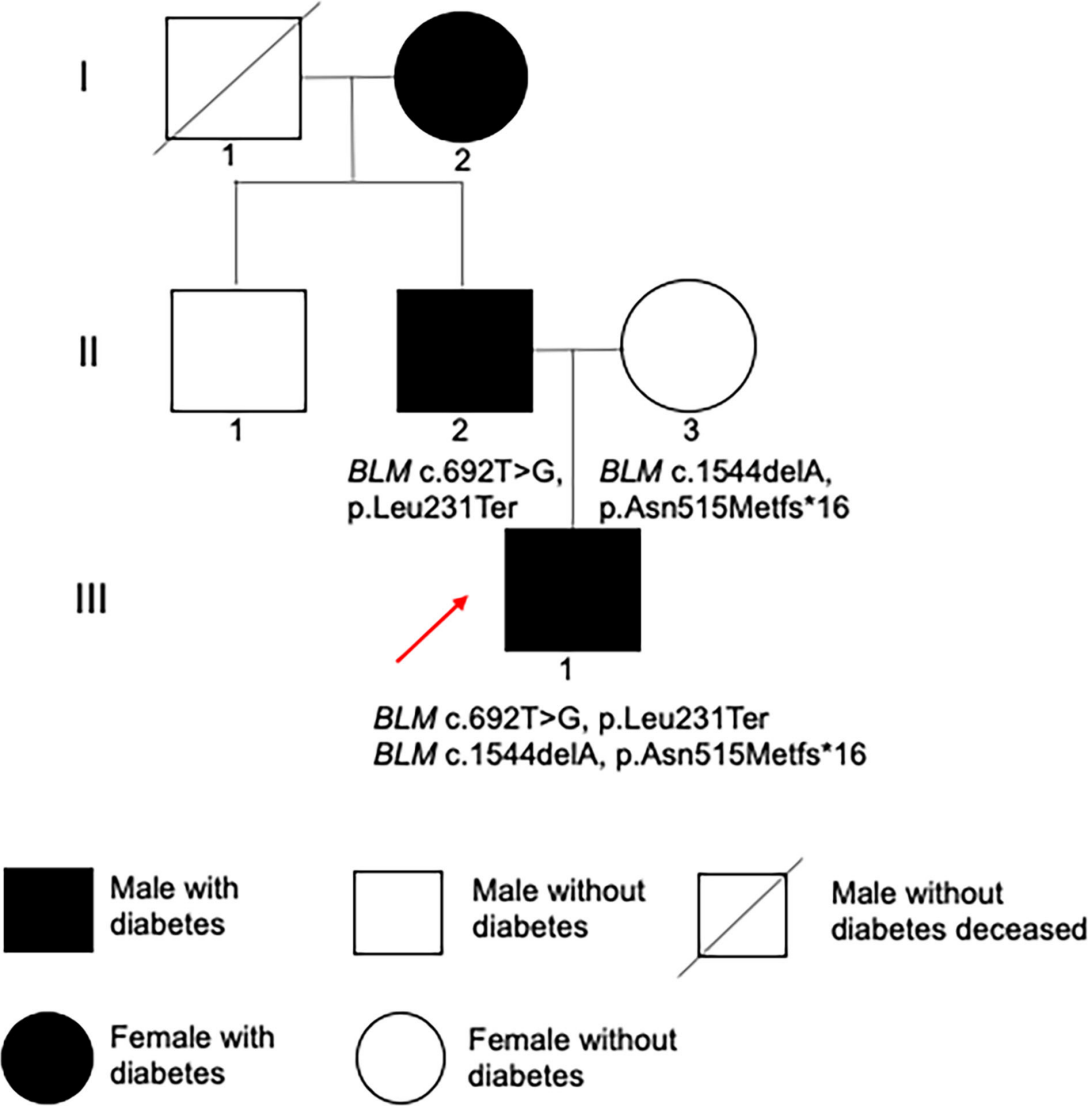
Family pedigree. Filled black symbols represent individuals with diabetes. The red arrow indicates the proband.

The proband had heterozygous *BLM* (BLM RecQ like helicase gene) variants (NM_000057.4), c.692T>G (p.Leu231*) and c.1544delA (p.Asn515Metfs*16) heterozygous variants, inherited from their parents, respectively (**Figures 3** and **4**). *BLM* c.1544delA (p.Asn515Metfs*16) has been reported to be pathogenic (1). *BLM* c.692T>G (p.Leu231*), which results in a truncated protein, was neither found in ExAC nor 1000G. The sequence variant was assigned to be pathogenic (PVS1+PP3+PP4) according to the guidelines of the American Society of Medical Genetics and Genomics (ACMG).

## Discussion

BS (OMIM 210900) is an autosomal recessive hereditary disease caused by a sequence variant in the *BLM* on chromosome 15q26.1, which encodes RecQ helicase (RECQL3). RecQ helicase is an important DNA helicase during DNA replication and repairmen, maintaining DNA stability when DNA double strands are unfolded. BS is rare, with no more than 300 cases reported globally and about one-third of the patients are German Jews (1). At present, only one family of BS without diabetes is reported in China (2). Carbohydrate and lipid metabolism, as well as growth and development disorder, may be involved in BS. However, a detailed description of endocrine system involvement from an endocrinologist perspective is scarce.

BS is a rare cause of diabetes. According to the BS registration system (https://www.ncbi.nlm.nih.gov/books/NBK1398/), 16% of patients with BS have T2D. However, different from typical T2D, patients with BS developed diabetes at a younger age and have a lower BMI. The pathogenesis of diabetes in patients with BS is unclear. Diaz et al. suggested that insulin resistance plays an important role in the glucose metabolism of BS (3). In our case, a high dose of insulin failed to control blood glucose, which indicated that the patient had significant insulin resistance. However, it is worth noting that, similar to our patient, none of the BS patients had acanthosis nigricans. As we have known, insulin resistance is related to abnormal secretion of adipokines (1). His leptin level was 0.5 ng/mL, which suggested that abnormal adipokines secretion might be involved in the pathogenesis of his diabetes. However, unlike diabetes resulted from lipoatrophy, no significant dyslipidemia was presented in our patient, and quantification and distribution of fat were still within the normal range. It is also worth noting that our patient occurred ketosis at the onset of diabetes, and his islet function was poorer compared with classical T2D, suggesting that islet failure may also be involved in patients with BS. Our finding supports the conclusion from Kondo et al. that insulin-dependent diabetes can also be developed in early adulthood in BS (4). In a word, insulin resistance and islet β-cell dysfunction may both be involved in the development of diabetes in patients with BS. By combining metformin and pioglitazone based on insulin, our patient’s blood glucose was well controlled. Moreover, the risk of cancers in BS is high, resulting in their short lifetime. As metformin is beneficial for reducing hyperglycemia-induced genome instability (5), which might make it count in improving clinical outcomes of BS patients. In addition to diabetes, patients with BS can also have other endocrine abnormalities. BS patients often come to an endocrinologist because of their short stature. It is extremely important for an endocrinologist to recognize BS and then avoid GH treatment. As for patients with hypogonadism or azoospermia, BS should be included in the differential diagnosis.

*BLM* is the only known gene that causes BS. For now, less than 80 pathogenic sequence variants have been identified (http://www.hgmd.cf.ac.uk/ac/gene.php?gene=BLM). The homozygote at the 2281 position of the 6 bp deletion and the 7 bp insertion is referred to as *blm^Ash^*. The presence of *blm^Ash^* in over 95% of the BS chromosomes is due to the co-founder’s ancestry inheritance. The *BLM* sequence variants so far identified have also suggested additional founder sequence variants. Among 64 different sequence variants that were reported by German et al, 19 were recurrent (6). No recurrent sequence variant has been found in Chinese BS. In the only reported Chinese BS pedigree, the proband carried a reported heterozygous sequence variant c.772_773delCT and a new sequence variant c.959 +2T>A (2). In our proband, *BLM* c.1544delA has been reported, while *BLM* c.692T>G is a new sequence variant. Our research expanded the BS genetic spectrum. However, functional tests *in vivo* and *vitro* are necessary to further establish pathogenicity of the new variant, and sequencing of genomic DNA in more family members may help to deepen our understanding of *BLM* variants.

In conclusion, we present the first case of BS with diabetes in the Chinese population, indicating BS may be one rare cause of diabetes in the Chinese population. In this study, the sequence variant of *BLM* c.1544delA has been reported, while *BLM* c.692T>G is a new sequence variant, which may expand the spectrum of *BLM* variants.

## Ethics Statement

This study was approved by the Ethics Committee of Peking Union Medical College Hospital. The patients/participants provided their written informed consent to participate in this study. Written informed consent was obtained from the individual(s) for the publication of any potentially identifiable images or data included in this article.

## Author Contributions

All the authors have contributed significantly. MD collected the clinical data, wrote the manuscript. RJ summarized the relevant literature. KF and XX give suggestions about clinical investigations. All the work was done under the instructions of MY. All authors contributed to the article and approved the submitted version.

## Funding

The work is supported by the National Key Research and Development Program of China (2018YFC2001100, 2016YFC0901500) and Chinese Academy of Medical Sciences Innovation Fund for Medical Sciences (CIFMS2017-I2M-1-008).

## Conflict of Interest

The authors declare that the research was conducted in the absence of any commercial or financial relationships that could be construed as a potential conflict of interest.
